# The prevalence of diabetes distress and its association with glycaemia in young people living with insulin‐requiring‐diabetes in a regional centre in Australia

**DOI:** 10.1111/jpc.16221

**Published:** 2022-10-07

**Authors:** Ciara Stapleton, Elizabeth Watkins, Matthew J L Hare, Francesca Timms, Anna J Wood, Angela Titmuss

**Affiliations:** ^1^ Paediatric Department, Division of Women, Children and Youth Royal Darwin Hospital Darwin Northern Territory Australia; ^2^ Endocrinology Department, Division of Medicine Royal Darwin Hospital Darwin Northern Territory Australia; ^3^ Wellbeing and Preventable Chronic Diseases Division Menzies School of Health Research, Charles Darwin University Darwin Northern Territory Australia

**Keywords:** adolescents, children, diabetes, distress, glycaemia, parents

## Abstract

**Aim:**

Emotional responses, such as feeling overwhelmed with diabetes‐related treatment, burnt‐out and anxiety, are known as ‘diabetes distress’. This study aimed to determine diabetes distress among children, adolescents and parents/carers managing insulin‐requiring diabetes in a regional Australian setting, and to assess association with glycaemia.

**Methods:**

All children, adolescents and their parents/carers attending a regional hospital outpatient diabetes clinic between March 2018 and June 2019 were invited to complete a validated child, adolescent or parent/carer diabetes distress questionnaire. Demographics and time‐matched clinical data were obtained from hospital records. A cross‐sectional analysis was performed.

**Results:**

A total of 43 young people and 30 parents/carers completed a diabetes distress questionnaire during the study period. Diabetes distress was common, with 63% of young people and 67% of parents/carers nominating at least one serious concern. After adjustment for potential confounding factors, higher glycaemia (HbA_1c_%) was associated with higher distress scores among both young people (ß 6.2, 95% confidence interval (CI): 3.2–9.2, *P* < 0.001) and carers/parents (ß 5.6, 95% CI:1.5–9.8, *P* < 0.001). Diabetes distress did not differ by child age, duration of diagnosis or mode of insulin administration. For children, adolescents and carers, ‘serious concerns’ most commonly related to the impact of diabetes upon family and peer relationships.

**Conclusions:**

Diabetes distress was common and associated with sub‐optimal glycaemia. Routine screening for diabetes distress should be considered in paediatric services. Development of strategies to minimise diabetes distress for youth and families is required.

## What is already known on this topic

1. Living with a long‐term condition such as diabetes yields a significant burden, as well as creating emotional hardship for children, adolescents and their parents/carers. This burden and the distress associated is termed ‘diabetes distress’.
2. Higher diabetes distress for adults with diabetes is associated with elevated glycaemia, less adherence to recommended management strategies and adverse health outcomes.
3. Glycaemia is an important marker of diabetes treatment success and predicts future health outcomes. In Australia, only one in four young people with diabetes meet recommended glycaemia targets, with diabetes distress possibly contributing.

## What this paper adds

1. This study identified moderate to severe levels of diabetes distress among a cohort of regionally located Australian children, adolescents and their parents/carers.
2. This study contributes to the growing collection of international evidence that reports on greater diabetes distress being associated with elevated glycaemia among the paediatric and adolescent population with insulin‐requiring diabetes.
3. This study provides insight into the emotional experience of diabetes for children, adolescents and their parents/carers in the Australian context.

Living with a long‐term condition such as diabetes can be demanding, relentless and overwhelming for children, adolescents and their parents/carers.^1^ In order to maintain optimal blood glucose levels, people with diabetes and carers need to adopt multiple complex daily adherence behaviours.^2^, ^3^ Common emotional responses include feeling burdened with treatment tasks, guilt regarding imperfect management, worries about the future, and generally feeling burned out. Such emotional responses are known as ‘diabetes distress.^1^ Increasing diabetes distress in adults is associated with elevated glycaemia, decreased adherence to recommended management strategies, and poorer health outcomes.^4^

The Problem Areas In Diabetes (PAID) questionnaire^5^ has been used in the adult population to identify diabetes distress and enable pathways towards minimising distress and improving diabetes outcomes.^6^ Adaptations measuring such concerns among paediatric and adolescent populations with diabetes have only recently been developed,^7^, ^8^ and international guidelines recommend regular routine assessment of diabetes distress.^9^

Our study aimed to assess diabetes distress among children and adolescents with insulin‐requiring diabetes (type 1 and type 2 diabetes) and their carers, as well as the association of diabetes distress with glycaemia. The prevalence of diabetes distress among Australian children, adolescents, and their parents/carers is unknown, with only one previous study exploring diabetes distress in Australian adolescents.^10^ Our study also provides a unique opportunity to explore diabetes distress in the regional Australian context. Regional centres typically have restricted access to specialised support or mental health services.^11^, ^12^ Understanding diabetes distress in this context is critical in facilitating pathways to improve mental health and diabetes outcomes in childhood and adolescence, as well as promoting lifelong positive self‐management.

## Methods

### Participants

All young people (children and adolescents) with insulin‐requiring diabetes and their parents/carers attending paediatric diabetes appointments from March 2018 to June 2019 were invited to complete a diabetes distress questionnaire. Completion of the questionnaire was voluntary.

### Demographic and clinical information

The following variables were assessed among participating young people: age, sex, type of diabetes, duration of diabetes and treatment modality.

### Glycaemia

Haemoglobin A_1c_ (HbA_1c_) was measured as part of routine care. Trained staff obtained capillary blood samples from participants via finger prick and analysed using a calibrated Siemens DCA Vantage A1C Analyser.

### Diabetes distress measures

Four validated adaptations of the PAID scale^5^ were used to assess diabetes distress among the cohort; child (17‐item PAID‐C),^13^ adolescent ‘teen’ (20‐item PAID‐T),^14^ and two‐parent adaptations (21‐item P‐PAID‐C for parents/carers of children and 18‐item P‐PAID‐T for parents/carers of adolescents).^13^, ^14^ Participants rated the degree by which they felt upset or bothered by diabetes‐related situations over the past month on a Likert scale from 1 to 6 (1–2 ‘not a problem’, 3–4 ‘moderate problem’ and 5–6 ‘serious problem’). A sample of questions has been included in Table 1. Item scores were summed to form a total, with higher scores indicating greater diabetes distress. Analysis of total scores is the recommended means by which to interpret the questionnaires, with the total score representing the overarching construct of diabetes distress for each individual.^13^, ^14^ While each questionnaire utilised a consistent Likert scale (1–6), they differed in the total number of questions asked. In order to compare total scores between groups, questionnaire results were transformed so that the results were presented as a standardised percentage score (0–100), methodology consistent with previous studies.^15^, ^16^

**Table 1:** Example questions included in paediatric adapted PAID questionnaires

Identifying your Problem Areas in Diabetes – Child version (PAID‐C)
Feeling upset when I see high blood sugar numbers on my meter.
Feeling that I must be perfect in caring for my diabetes.
Worrying about having a low blood sugar during a sports activity.
Identifying your Problem Areas in Diabetes – Parent of child version (P‐PAID‐C)
Feeling overwhelmed by my child's diabetes regimen.
Feeling constantly concerned about food and eating.
Worrying about the future and the possibility of my child developing serious complications.
Identifying your Problem Areas In Diabetes – Teen version (PAID‐T)
Feeling sad when I think about having and living with diabetes.
Feeling discouraged or defeated when I see high blood sugar results on my meter.
Feeling that my friends or family act like ‘diabetes police’ (e.g. nag about eating properly, checking blood sugars, not trying hard enough).
Identifying your Problem Areas in Diabetes – Parent of teen version (P‐PAID‐T)
Feeling that I am often failing with managing my child's diabetes regime.
Feeling discouraged or defeated when I see high blood sugar results on my child's meter.
Worrying that my child will miss or skip blood sugar checks.

In this clinical setting, it was common for the treating team to look not only at the total score, but also to scan for individual questions or themes causing severe distress, even when the total score described low‐moderate levels of overall distress. As such, in addition to the total score, the distress measures were assessed in this study for frequency of serious concerns (score 5–6) across the whole questionnaire, as well as within identified sub‐themes of diabetes distress. While these questionnaires utilise variable age‐appropriate language, they consistently feature questions addressing four specific themes: negative emotions, daily demands, long‐term outcomes, and the impact of diabetes on relationships.^14^, ^15^ Frequency of ‘serious concerns’ was compared to assess for differences in these themes of diabetes distress among children, adolescents and parents/carers.

When diabetes distress was identified, the diabetes team would acknowledge areas of distress, engage in therapeutic listening, and invite the family and child to discuss potential solutions/strategies. At times, referrals to primary health‐care services, private psychology, and/or Child and Adolescent Mental Health Services/Headspace were also needed.

### Statistical analysis

Descriptive statistics are reported as number and proportion (%), mean (±standard deviation) when normally distributed, or median and IQR when not normally distributed. Associations of clinical and demographic factors with diabetes distress scores among young people were assessed using crude and adjusted linear regression models. Sex, mode of insulin delivery, age, HbA_1c_ and duration of diabetes were included *a priori* in the multivariable model. A sensitivity analysis used a stepwise model‐building approach, including variables in the multivariable model only if they were associated with diabetes distress in crude models. There was no meaningful difference in the results. Determination of significance was based on *P* value < 0.05 and clinical relevance of effect sizes. Analyses were undertaken in Stata V15.1 (StataCorp, TX).

The study was approved by the Human Research Ethics Committee of the Northern Territory Department of Health and Menzies School of Health Research (HREC 2018‐3247).

## Results

### Sample characteristics

A total of 43 (12 children and 31 adolescents) out of 45 eligible young people with insulin‐requiring diabetes attending a paediatric diabetes clinic appointment within the study period, completed a diabetes distress questionnaire (96% response rate). A total of 30 (11 children, 19 adolescents) young people had a time‐matched questionnaire completed by a parent/carer, resulting in 73 unique individuals included in this study.

Characteristics of the 43 young people are outlined in Table 2. The majority of young people had a diagnosis of type 1 diabetes (*n* = 40, 93%). Median diabetes duration was 7.0 (2–9) years and 23 (53%) young people were using a continuous subcutaneous insulin infusion pump. Median HbA_1c_ was 68 mmol/mol (8.4%). A total of 11 young people (26%) had an HbA_1c_ equal or below the Australian recommended target level of 58 mmol/mol (7.5%).^17^

**Table 2:** Study group characteristics and distress measures

Measure	All young people (*n* = 43)	Children (*n* = 12)	Adolescents (*n* = 31)
Age (years)	14.1 (12.6–16.2)	11.8 (10.6–12.7)	15.2 (13.3–17.2)
Female	19 (44%)	5 (42%)	14 (45%)
Aboriginal and/or Torres Strait Islander ethnicity	4 (9%)	0 (0%)	4 (13%)
Type 1 diabetes	40 (93%)	11 (92%)	29 (94%)
Diabetes duration (years)	7 (2–9)	6 (1–9)	7 (2–9)
Type of insulin therapy – CSII	23 (53%)	6 (50%)	14 (45%)
Type of insulin therapy – MDI	20 (47%)	6 (50%)	17 (55%)
Mean HbA_1c_ (NGSP %)	8.8 (1.9)	8.0 (1.4)	9.1 (2.0)
Mean HbA_1c_ (mmol/mol)	73 (20)	64 (15)	76 (22)
Median HbA_1c_ (NGSP %)	8.4 (7.5–9.8)	8.0 (6.8–8.5)	8.6 (8.0–10.0)
Median HbA_1c_ (mmol/mol)	68 (58–84)	64 (51–69)	70 (64–86)
Young person's PAID *d* total diabetes distress score (percentage 0–100)†	38 (21)	32 (17)	41 (22)
Young person nominating at least 1 statement as causing severe diabetes distress (score 5–6)	27 (63%)	7 (58%)	20 (65%)
	All parents/carers (*n* = 30)	Parents/carers of children (*n* = 11)	Parents/carers of adolescents (*n* = 19)
Parent/carer's PAID total diabetes distress score (percentage 0–100)#	43 (18)	43 (14)	44 (21)
Parent/carer nominating at least 1 statement as causing severe diabetes distress (score 5–6)	20 (67%)	8 (73%)	12 (63%)

† Child (PAID‐C) and adolescent (PAID‐T) adaptations of the PAID diabetes distress questionnaire use a consistent Likert scale (1–6) but differ in total number of questions asked. Questionnaire results were therefore transformed and results are presented as a standardised percentage score (0–100).

# Parent/carer of child (P‐PAID‐C) and parent/carer of adolescent (P‐PAID‐T) adaptations of the PAID diabetes distress questionnaire use a consistent Likert scale (1–6) but differ in total number of questions asked. Questionnaire results were therefore transformed and results are presented as a standardised percentage score (0–100).

N.B. Data are *n* (%), mean (SD) or median (IQR). CSII, continuous subcutaneous insulin infusion; HbA_1c_, glycated haemoglobin A_1c_; MDI, multiple daily injection; PAID, Problem Areas In Diabetes Questionnaire.

### Diabetes distress

The mean diabetes distress score percentage for children and adolescents combined was 38 (SD 21) and 43 for parents/carers combined (SD 18). Mean diabetes distress scores for each subgroup are presented in Table 2. Diabetes distress was a common experience, with 7 children (58%), 20 adolescents (65%), 8 parents/carers‐of‐children (73%) and 12 parents/carers‐of‐teens (63%) nominating at least one serious concern (score of 5–6 on one or more questions). Across all groups, there was an average of three serious concerns (range 0–19, SD 4.2).

**Fig. 1:**
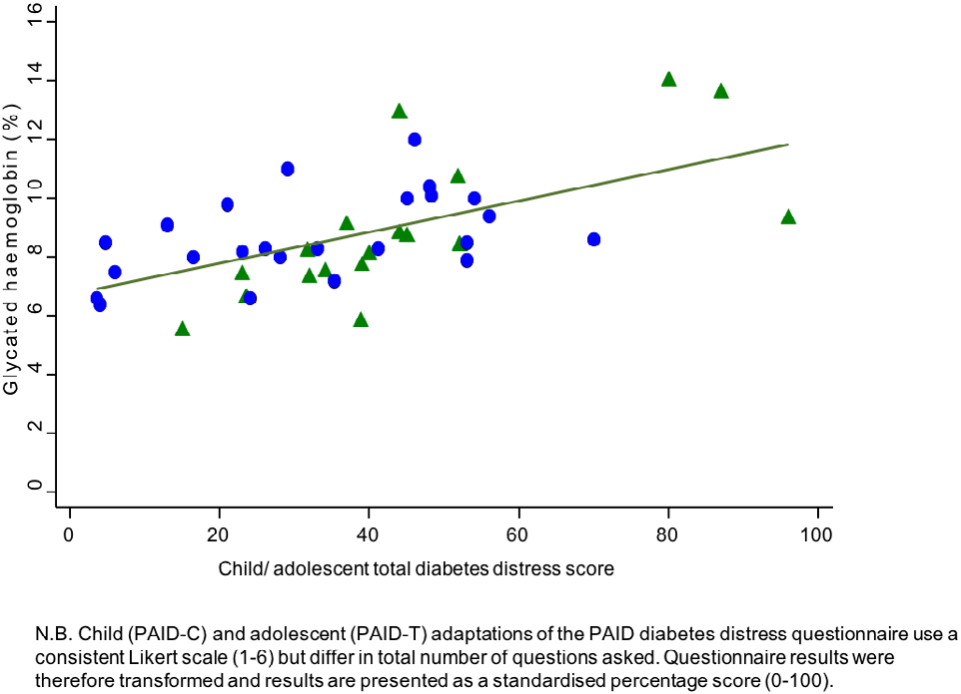
Total diabetes distress score for each child/adolescent, correlated with glycaemia. (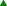) Female and (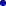) Male.

**Fig. 2:**
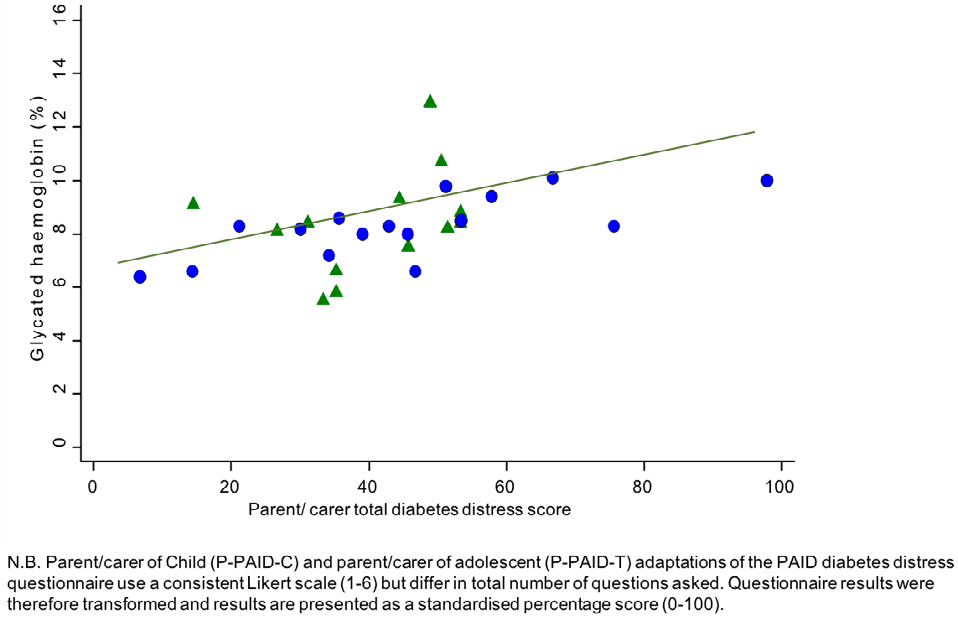
Total diabetes distress score for each parent/carer, correlated with the young person's glycaemia. (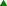) Female child/youth and (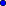) Male child/youth.

Associations of clinical and demographic factors with diabetes distress scores among young people were assessed (Table 3). Higher diabetes distress scores were associated with higher HbA_1c_ (Figs. 1 and 2). Diabetes distress scores among children and adolescents were associated with HbA_1c_, such that for each percentage increase in HbA_1c_, the diabetes distress score percentage was 6.2 points higher (95% confidence interval (CI): 3.2–9.2, *P* < 0.001). Diabetes distress scores among parents/carers were associated with HbA_1c_ in young people, such that for each percentage increase in HbA_1c_, diabetes distress score percentage was 5.6 points higher (95% CI: 1.5–9.8, *P* = 0.010). These relationships persisted after adjustment for potential confounding factors including young person's sex and age, duration of diabetes and mode of insulin delivery.

**Table 3:** Associations of clinical and demographic factors with diabetes distress scores among young people measured using crude and multivariable‐adjusted regression models

	Unadjusted associations with diabetes distress score in univariable models		Adjusted associations with diabetes distress score in multivariable model	
	β (95% CI)	*P* value	β (95% CI)	*P* value
Female (vs male)	13.0 (0.6–25.5)	0.041	11.4 (1.1–21.8)	0.032
CSII (vs insulin pens)	−7.0 (−19.9–5.8)	0.276	−2.7 (−13.6–8.2)	0.615
Age (years)	2.1 (−0.3–4.6)	0.088	0.03 (−2.2–2.2)	0.980
HbA_1c_ (NGSP%)	6.5 (3.6–9.3)	<0.001	6.2 (3.2–9.2)	<0.001
Diabetes duration (years)	0.8 (−0.8–2.4)	0.316	0.8 (−0.5–2.1)	0.214

N.B. Multivariable model included all listed variables (sex, mode of insulin delivery, age, HbA1c and diabetes duration). CSII, continuous subcutaneous insulin infusion; HbA_1c_, haemoglobin A1c.

Female young people had higher total distress scores than male (female mean 45.5 (SD 21.3) vs. male mean 32.6 (SD 19.0), *P* = 0.04). This relationship persisted after adjustment for potential confounding factors.

No significant difference in the distress experience of caring for a female child, compared to a male child with insulin‐requiring diabetes, was seen for parental/carer distress scores.

No significant difference was found between distress scores of those managing diabetes using a continuous subcutaneous insulin infusion pump, compared to those using multiple daily injection insulin regimens (*P* = 0.615). Distress scores for young people and their parents/carers were not associated with the young person's age or duration since diabetes diagnosis.

### Diabetes distress themes

For children, adolescents and their parents/carers, the theme that most frequently prompted a ‘serious concern’ score (Likert 5–6) related to the impact of diabetes upon relationships with the child or parent, as well as relationships with peers (Table 4). For children, long‐term outcomes, and daily demands were infrequently nominated as sources of ‘serious’ distress. For adolescents, and parents of both children and adolescents, all themes appeared to be potential key sources of diabetes distress.

**Table 4:** Frequency of reported serious concerns (score of 5–6 on a relevant question) according to theme

Themes of diabetes distress	Number (%)			
	Children (*n* = 12)	Parents/carers of children (*n* = 11)	Adolescents (*n* = 31)	Parents/carers of adolescents (*n* = 19)
Negative emotions	3 (25%)	5 (45%)	11 (35%)	4 (21%)
Daily demands	1 (8%)	5 (45%)	13 (42%)	7 (37%)
Impact upon relationships	7 (58%)	6 (55%)	15 (48%)	10 (53%)
Distress due to worry related to long‐term outcomes	1 (8%)	3 (27%)	9 (29%)	7 (37%)

## Discussion

To the best of our knowledge, this is the first published study exploring the integration of a diabetes distress questionnaire within routine care at an Australian paediatric diabetes service. Our study assesses diabetes distress within an Australian non‐tertiary context, contributing to knowledge regarding factors affecting the chronic condition experience by youth with diabetes and their families. First, we report significant diabetes distress among the majority of children, adolescents, and their parents/carers. Second, we report limited demographic factors that can be used to predict higher risk of diabetes distress. Third, we report an association between diabetes distress and glycaemia, with higher distress scores associated with higher glycaemia. Finally, we report differences in experiences and thematic categories of distress between children, adolescents and their parents/carers.

Our study demonstrated a high proportion of children, adolescents and carers experience diabetes distress, consistent with previous studies.^8^, ^10^, ^15^, ^16^, ^18^ The mean percentage scores in our study ranged from 32 for children, to 44 for parents‐of‐adolescents, with a maximum possible percentage score being 100 (i.e. scoring 6/6 on all proposed questions). There is no established threshold score for defining diabetes distress in a paediatric cohort with these questionnaires. However, other similar, though not identical, PAID questionnaires have used various thresholds between 34 and 50/100 to identify ‘severe distress’.^8^, ^15^, ^16^, ^18^, ^19^, ^20^ The mean distress scores reported by our cohort suggest that a large portion are experiencing moderate to severe diabetes distress.

In addition to high overall distress, 58% of children, 65% of adolescents, 73% of parents‐of‐children and 63% of parents‐of‐adolescents, declared at least one serious concern (item score 5–6) in our study. Across all groups, there was a mean of three serious concerns (range 0–19, SD 4.2). This finding is consistent with an American study where 54% of adolescents declared at least one serious concern, with a mean of 3.3 (SD 4.8) serious concerns.^21^ The finding of elevated distress scores in all subgroups of our study reinforces the need to screen for distress across all groups, and to optimise the diabetes team's approach to these issues.

Our study demonstrated limited significant associations between demographic characteristics and reported diabetes distress scores. While female participants reported higher diabetes distress than males in our cohort, the majority of published studies report no gender association.^8^ The observed gender difference in our cohort requires further assessment, but could reflect the small sample size, the potential impact of strong gender stereotypes on mental health disclosure in our community, or other unknown factors. In our cohort, factors such as the young person's age, diabetes diagnosis duration, or mode of insulin delivery did not appear to significantly impact upon diabetes distress. This aligns with several international studies which also assessed these associations.^8^, ^20^, ^21^ Given that diabetes distress scores did not significantly differ by demographic variables, our study reinforces that care providers cannot reliably predict who may be experiencing diabetes distress. It is important to consider this possibility in all young people and their parents/carers.

We report higher glycaemia to be associated with higher diabetes distress for both the young person as well as for their parent/carer. This finding is consistent with the limited number of studies^8^, ^10^, ^18^ and extends this knowledge to the Australian regional paediatric context. This association may be influenced by several factors, with psychosocial difficulties complicating the self‐care activities required in diabetes management. In addition, suboptimal HbA_1c_, with associated elevated and fluctuating blood glucose levels, can further negatively impact psychosocial functioning.^22^ Children and adolescents attending our service had a median HbA_1c_ of 68 mmol/mol (8.4%), with only one in four achieving HbA_1c_ within recommended targets. These values align with those in a recent nationwide Australian audit in which 27% of young people were achieving target glycaemia and the median HbA_1c_ was 65 mmol/mol (8.1%).^17^ Despite recent advancements in insulin‐delivery and glucose‐monitoring technologies, glycaemic outcomes remain suboptimal for the majority of young people with diabetes^17^, ^23^ and diabetes distress may represent an important risk factor and/or perpetuator of this issue.

Finally, this study identified variations in the themes most typically causing distress between young people and their parents/carers. In our study, questions relating to impact upon relationships (including parental relationships) were most frequently reported by children as a source of ‘serious distress’. This potentially reflects child development, where the burden of daily treatment tasks is experienced through their relationship with a carer/parent rather than independent management. Adolescents and parents/carers reported ‘serious distress’ more evenly across the four themes (experiencing negative emotions, facing daily demands, impact upon relationships and worry related to long‐term outcomes). While adolescents frequently acknowledged the impact on relationships as a source of serious distress (48%), worry regarding long‐term outcomes was also a key contributor (29%). This is a somewhat unexpected finding as typical adolescent developmental models highlight a heightened focus on peer relationships, independence and a tendency towards impulsivity and risk‐taking rather than long‐term planning.^24^ For adolescents in our study, this finding may suggest differing psychological development of young people with chronic conditions from their non‐affected peers. Further exploration of diabetes distress themes is required to better understand the distress experience of young people and parents/caregivers.

People living in regional, rural and remote areas of Australia generally experience poorer health outcomes, and ultimately have a shorter life expectancy from birth compared to those living in major cities.^25^ Likely contributors to this include increased health risk factors (such as obesity, smoking, diet and physical activity), as well as reduced access to health‐care services. A recent survey of the paediatric diabetes workforce across Australia and New Zealand highlighted the low numbers of specialist medical and allied health staff particularly in regional centres,^12^ a finding which mirrors the workforce available at the site of this study. This context may lead to higher rates of diabetes distress in regional settings compared to tertiary centres though no data is available to validate this. However, we note that participants in our study have similar HbA_1c_ to Australian tertiary centres.^17^ Understanding the chronic disease experience for young people and their families living in regional centres of Australia is essential in order to better target efforts to address these health disparities.

The strengths of this study include the use of an objective measure of diabetes distress, and data capture occurring during routine clinical care, with glycaemia and diabetes distress both measured at the same time point. Detailed demographic and clinical information was also available for all young people. The high rate (96%) of voluntary questionnaire completion strengthens the reliability of the findings. The study reports on the use of a standardised diabetes distress questionnaire for both children, adolescents and their parents/carers for the first time within routine care in Australia, thus expanding our understanding of the diabetes experience.

Limitations of this study include its cross‐sectional nature with a relatively small cohort. A sample size was not calculated for the purpose of addressing differences in glycaemic or distress outcomes, and as such this study may have been underpowered for some of the comparisons presented. Furthermore, some of the reported findings, such as the scale of association between diabetes distress and HbA_1c_, are novel, with no similar studies to corroborate the significance of the findings. The causality or direction of the relationship between diabetes distress and HbA_1c_ could not be assessed and there was limited assessment of potential confounding factors. Furthermore, generalisability of the findings to other diabetes services in Australia and internationally may be limited given the characteristics of this regional centre. However, there are likely commonalities of experience with many regional centres, and it is important for these services to consider how diabetes distress could be minimised in their cohort, particularly when limited mental health services may be available.

## Conclusion

Our study demonstrates that children and adolescents with insulin‐dependent diabetes, and their carers, attending a non‐tertiary regional health service, experience high levels of diabetes distress, and that this distress is associated with elevated glycaemia. Our findings support screening for diabetes distress among all children and adolescents with diabetes, as well as their parents/carers, especially when glycaemia is suboptimal. Our study provides evidence to support international guidelines^8^ which recommend assessing mental health factors such as diabetes distress at regular intervals. Health services caring for young people and families with diabetes should consider their own policy and practices, reflecting upon how chronic disease‐related mental health issues may be assessed and supported in their context. This study has highlighted the need for further research in this field, particularly research that might improve our understanding of potential causal and temporal relationships between diabetes distress and glycaemia, and research to assess the interaction of parent and child distress experiences. Further work evaluating the effectiveness of varied diabetes distress minimisation strategies is also needed.

## References

[1] Hilliard ME , Yi‐Frazier JP , Hessler D , Butler AM , Anderson BJ , Jaser S . Stress and A_1c_ among people with diabetes across the lifespan. Curr. Diab. Rep. 2016; 16: 67. 27287017 10.1007/s11892-016-0761-3 PMC4936828

[2] Lansing AH , Berg CA . Adolescent self‐regulation as a foundation for chronic illness self‐management. J. Pediatr. Psychol. 2014; 39: 1091–6. 25214646 10.1093/jpepsy/jsu067 PMC4201765

[3] Henríquez‐Tejo R , Cartes‐Velásquez R . Psychosocial impact of type 1 diabetes mellitus in children, adolescents and their families. Literature review. Rev. Chil. Pediatr. 2018; 89: 391–8. 29999147 10.4067/S0370-41062018005000507

[4] Peyrot M , Rubin RR , Lauritzen T , Snoek FJ , Matthews DR , Skovlund SE . Psychosocial problems and barriers to improved diabetes management: Results of the Cross‐National Diabetes Attitudes, Wishes and Needs (DAWN) Study. Diabet. Med. J. Br. Diabet. Assoc. 2005; 22: 1379–85. 10.1111/j.1464-5491.2005.01644.x 16176200

[5] Polonsky WH , Anderson BJ , Lohrer PA *et al*. Assessment of diabetes‐related distress. Diabetes Care 1995; 18: 754–60. 7555499 10.2337/diacare.18.6.754

[6] Hendrieckx C , Halliday J , Beeney LJ , Speight J . Diabetes and Emotional Health: A Handbook for Health Professionals Supporting Adults with Type 1 or Type 2 Diabetes. Canberra: National Diabetes Services Scheme; 2016. 10.2196/15007 PMC7060499 32130112

[7] Hilliard ME , De Wit M , Wasserman RM *et al*. Screening and support for emotional burdens of youth with type 1 diabetes: Strategies for diabetes care providers. Pediatr. Diabetes 2018; 19: 534–43. 28940936 10.1111/pedi.12575 PMC5862727

[8] Hagger V , Hendrieckx C , Sturt J , Skinner TC , Speight J . Diabetes distress among adolescents with type 1 diabetes: A systematic review. Curr. Diab. Rep. 2016; 16: 9. 26748793 10.1007/s11892-015-0694-2

[9] Delamater AM , de Wit M , McDarby V *et al*. ISPAD clinical practice consensus guidelines 2018: Psychological care of children and adolescents with type 1 diabetes. Pediatr. Diabetes 2018; 19: 237–49. 30058247 10.1111/pedi.12736

[10] Hagger V , Hendrieckx C , Cameron F , Pouwer F , Skinner TC , Speight J . Diabetes distress is more strongly associated with HbA_1c_ than depressive symptoms in adolescents with type 1 diabetes: Results from Diabetes MILES Youth‐Australia. Pediatr. Diabetes 2018; 19: 840–7. 29383803 10.1111/pedi.12641

[11] Perry L , Lowe JM , Steinbeck KS , Dunbabin JS . Services doing the best they can: Service experiences of young adults with type 1 diabetes mellitus in rural Australia. J. Clin. Nurs. 2012; 21: 1955–63. 22672458 10.1111/j.1365-2702.2011.04012.x

[12] de Bock M , Jones TW , Fairchild J , Mouat F , Jefferies C . Children and adolescents with type 1 diabetes in Australasia: An online survey of model of care, workforce and outcomes. J. Paediatr. Child Health 2019; 55: 82–6. 30051636 10.1111/jpc.14122

[13] Evans MA , Weil LEG , Shapiro JB *et al*. Psychometric properties of the parent and child problem areas in diabetes measures. J. Pediatr. Psychol. 2019; 44: 703–13. 30920628 10.1093/jpepsy/jsz018 PMC6573474

[14] Shapiro JB , Vesco AT , Weil LEG , Evans MA , Hood KK , Weissberg‐Benchell J . Psychometric properties of the problem areas in diabetes: Teen and parent of teen versions. J. Pediatr. Psychol. 2018; 43: 561–71. 29267939 10.1093/jpepsy/jsx146 PMC6454555

[15] Lohiya NN , Kajale NA , Lohiya NN , Khadilkar VV , Gondhalekar K , Khadilkar A . Diabetes distress in Indian children with type 1 diabetes mellitus and their mothers. J. Pediatr. Endocrinol. Metab. 2021; 34: 209–16. 33180040 10.1515/jpem-2020-0339

[16] Mianowska B , Fedorczak A , Michalak A *et al*. Diabetes related distress in children with type 1 diabetes before and during the COVID‐19 lockdown in spring 2020. Int. J. Environ. Res. Public Health 2021; 18: 8527. 34444274 10.3390/ijerph18168527 PMC8394974

[17] Phelan H , Clapin H , Bruns L *et al*. The Australasian diabetes data network: First national audit of children and adolescents with type 1 diabetes. Med. J. Aust. 2017; 206: 121–5. 28208043 10.5694/mja16.00737

[18] Toh ZQ , Koh SSL , Lim PK , Lim JST , Tam W , Shorey S . Diabetes‐related emotional distress among children/adolescents and their parents: A descriptive cross‐sectional study. Clin. Nurs. Res. 2021; 30: 311–21. 31387382 10.1177/1054773819867252

[19] Snoek FJ , Kersch NYA , Eldrup E *et al*. Monitoring of Individual Needs in Diabetes (MIND): Baseline data from the Cross‐National Diabetes Attitudes, Wishes, and Needs (DAWN) MIND study. Diabetes Care 2011; 34: 601–3. 21266654 10.2337/dc10-1552 PMC3041189

[20] Markowitz JT , Volkening LK , Butler DA , Laffel LMB . Youth‐perceived burden of type 1 diabetes: Problem areas in diabetes survey‐pediatric version (PAID‐Peds). J. Diabetes Sci. Technol. 2015; 9: 1080–5. 25910541 10.1177/1932296815583506 PMC4667338

[21] Weissberg‐Benchell J , Antisdel‐Lomaglio J . Diabetes‐specific emotional distress among adolescents: Feasibility, reliability, and validity of the problem areas in diabetes‐teen version. Pediatr. Diabetes 2011 Jun; 12: 341–4. 21443583 10.1111/j.1399-5448.2010.00720.x

[22] Tanenbaum ML , Kane NS , Kenowitz J , Gonzalez JS . Diabetes distress from the patient's perspective: Qualitative themes and treatment regimen differences among adults with type 2 diabetes. J. Diabetes Complications 2016; 30: 1060–8. 27217023 10.1016/j.jdiacomp.2016.04.023 PMC5792172

[23] Wood JR , Miller KM , Maahs DM *et al*. Most youth with type 1 diabetes in the T1D Exchange Clinic Registry do not meet American Diabetes Association or International Society for Pediatric and Adolescent Diabetes Clinical Guidelines. Diabetes Care 2013; 36: 2035–7. 23340893 10.2337/dc12-1959 PMC3687259

[24] Gibbons F , Kingsbury J , Gerrard M . Social‐psychological theories and adolescent health risk behavior. Soc. Personal. Psychol. Compass 2012; 6: 170–80.

[25] Australian Institute of Health and Welfare. Rural and Remote Health [Internet]. Canberra: Australian Institute of Health and Welfare; 2022 [cited 2022 August 25]. Available from: https://www.aihw.gov.au/reports/rural-remote-australians/rural-and-remote-health

